# Intraossärer Fremdkörper oder Osteom: Anspruchsvolle Differenzialdiagnose nach offener Radiusfraktur

**DOI:** 10.1007/s00113-021-01102-7

**Published:** 2021-12-03

**Authors:** K. Rau, F. Sauerwald, T. Grieser, L. Lisitano, E. Mayr, J. Plath

**Affiliations:** 1 Klinik für Unfallchirurgie, Orthopädie, Plastische und Handchirurgie, Universitätsklinikum Augsburg, Stenglinstraße 2, 86156 Augsburg, Deutschland; 2 Klinik für Diagnostische und Interventionelle Radiologie, Universitätsklinikum Augsburg, Stenglinstraße 2, 86156 Augsburg, Deutschland

**Keywords:** Epiphysenfugenverletzung, Offene Fraktur, Differentialdiagnostik, Sportverletzung, Mountainbike, Epiphyseal joint injury, Open fracture, Differential diagnosis, Sport injury, Mountain bike

## Abstract

Vorstellung eines 16-jährigen Patienten nach Fahrradsturz beim Mountainbiken 14 Tage nach primärer Versorgung nach offener Epiphysenverletzung. Metaphysär intraossär liegende Steine wurden bei anatomischer Reposition der Fraktur als nebenbefundliches Osteom fehlinterpretiert.

## Abstract (en)

Presentation of a 16-year-old male patient due to a cycling accident while mountain biking 14 days after primary treatment after open epiphyseal injury. Metaphyseal intraosseous stones within the anatomically reduced distal radius fracture were misinterpreted as an incidental osteoma.

## Falldarstellung

### Primärversorgung

Ein 16-jähriger Patient stellt sich in Begleitung seiner Mutter in der Hochschulambulanz der Universitätsklinik Augsburg vor. Vor 2 Wochen stürzte er in einem Bikepark in Österreich und zog sich eine Wunde sowie einen Bruch des rechten Handgelenks zu. Die medizinische Erstversorgung erfolgte in der Notaufnahme eines lokalen Krankenhauses mittels Wundnaht, Reposition im Aushang und Anlage einer Gipsschiene.

Die radiologischen Stellungs- und Wundkontrollen erfolgten heimatnah in einer niedergelassenen orthopädischen Praxis. Bei Fadenzug wurde klinisch der Verdacht auf einen lokalen Wundinfekt gestellt, und radiologisch wurden mögliche verbliebene Fremdkörper in den volaren Weichteilen als Ursache erkannt, weshalb der Patient an unsere Notaufnahme verwiesen wurde.

Der Patient sowie die Mutter wurden durch die vorbehandelnden Ärzte explizit darauf hingewiesen, dass es sich bei weiteren intraossär gelegenen röntgendichten Strukturen um gutartige Veränderungen handele, die keinerlei weiterer Diagnostik oder Therapie bedürfen.

Unsererseits wurde jedoch bei Verdacht auf intrafragmentär gelegene Fremdkörper eine CT-Diagnostik dringend angeraten.

### Befund

#### Klinischer Befund

**Figure 1:**
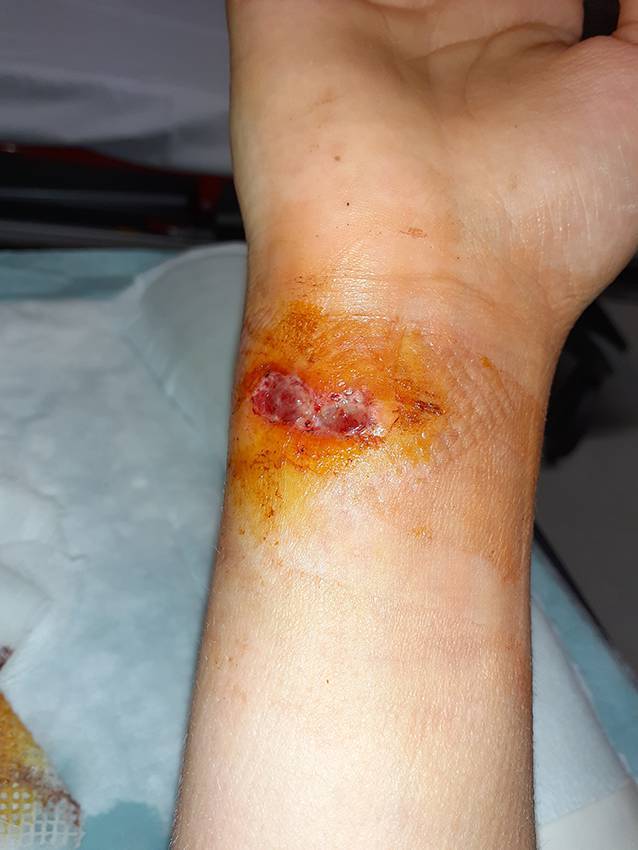

Auf der Beugeseite des rechten Handgelenks zeigte sich eine quer verlaufende, ca. 3 cm lange Wunde mit oberflächlicher Schürfung. Darunter war eine deutliche Fluktuation zu tasten. Eine signifikante Rötung oder Überwärmung bestand hingegen nicht. Auch die Flexorensehnen zeigten sich klinisch unauffällig. Sensomotorische Defizite bestanden ebenfalls nicht (Abb. 1).

Leukozytenzahl sowie CRP-Wert waren im Normbereich: (CRP 0,06 mg/dl [<0,05 mg/dl]; Leukozyten 3,66/nl [4,5–13/nl]).

#### Radiologischer Befund

**Figure 2:**
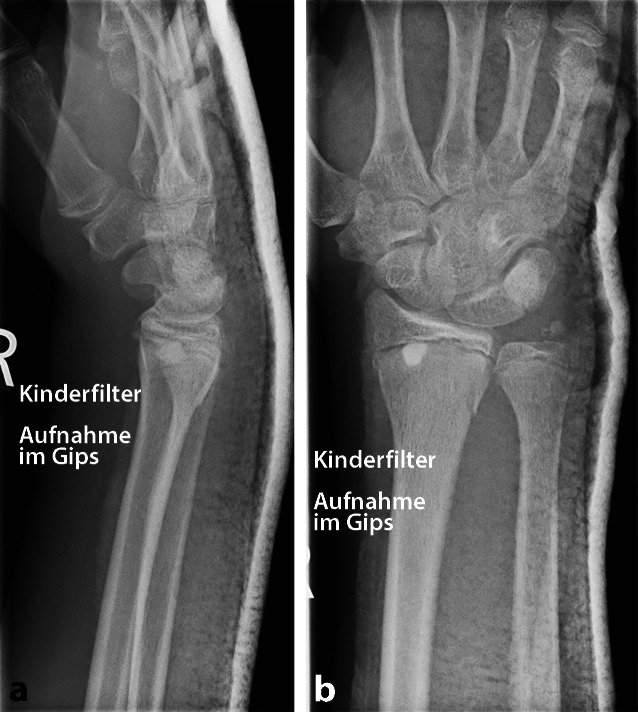

In den zuvor durchgeführten Röntgenaufnahmen zur radiologischen Stellungskontrolle (2, 6 und 12 Tage nach dem Trauma, je in 2 Ebenen) aus der orthopädischen Praxis war eine Aitken-I-Fraktur des distalen Radius mit zusätzlichem Abriss des Processus styloideus ulnae in anatomischer Stellung zu erkennen. Auffällig war eine rundliche metaphysär gelegene röntgendichte Struktur, welche sich in beiden Ebenen an die Epiphysenfuge anlegt. Ein Defekt im Sinne eines möglichen Fremdkörpereintritts im Knochen war nicht zu sehen. Des Weiteren zeigten sich zahlreiche kleinere röntgendichte Strukturen in den beugeseitigen Weichteilen (Abb. 2a, b).

**Figure 3:**
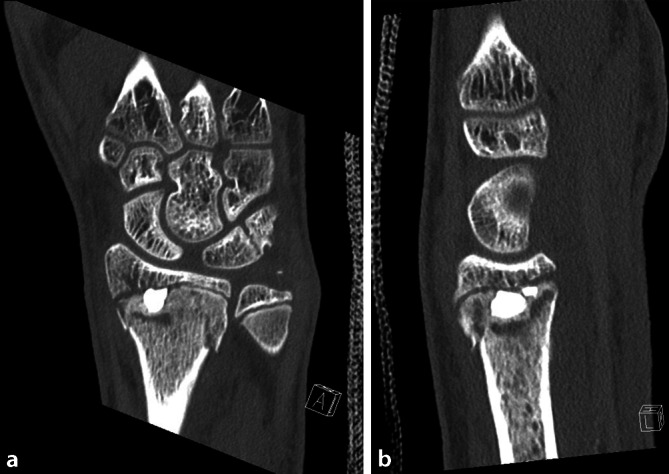

Das in unserer Notaufnahme durchgeführte CT erhärtete die Verdachtsdiagnose eines intraossären Fremdkörpers und zeigte einen zweiten kleineren intraossären Fremdkörper direkt volar des ersten (Abb. 3a, b).

#### Intraoperativer Befund

**Figure 4:**
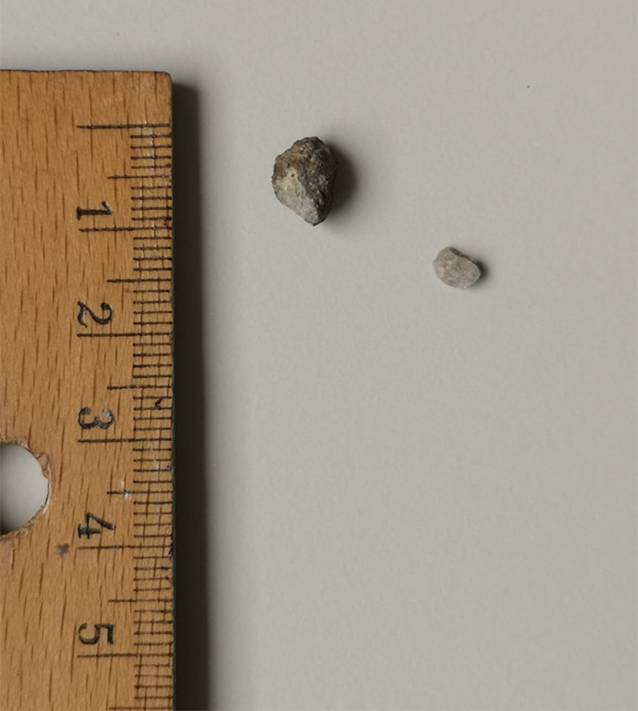

Bei Verdacht auf intraossär verbliebene Fremdkörper wurde die dringliche Indikation zu operativer Wundexploration, Débridement und Fremdkörperentfernung gestellt. Intraoperativ zeigten sich der M. pronator quadratus bereits eingeschmolzen sowie im Bereich der Metaphyse des distalen Radius eine beginnende Osteomyelitis. Es wurden Proben für die mikrobiologische Untersuchung entnommen. Es konnten 2 Steine aus der distalen Radiusmetaphyse geborgen werden (Abb. 4). Neben dem lokalen Débridement, der Probenentnahme und Spülung erfolgte die Einlage einer Septopal®-Kette (Zimmer Biomet Deutschland GmbH, Berlin, Deutschland) und ein Wundverschluss über einer Redon-Drainage. Eine additive Osteosynthese war bei ausreichender Stabilität der Fraktur nicht indiziert.

### Diagnose

Beginnende Osteomyelitis bei Z. n. 2°-offener Aitken-I-Fraktur des distalen Radius rechtsseitig mit interfragmentär gelegenen Steinen.

### Therapie und Verlauf

Die intraoperativ entnommenen mikrobiologischen Proben zeigten eine für das Erdreich typische Keimmischflora aus *Terrisporobacter glycolicus, Buttiauxella*-, *Achromobacter*-Spezies und *Clostridium tertium*. Alle Keime waren sensibel auf die postoperativ begonnene kalkulierte Antibiose mittels Ampicillin/Sulbactam (2000 mg/1000 mg).

Nach Entfernung der Septopal®-Ketten und 2-wöchiger i.v.-Antibiose im Rahmen des stationären Aufenthalts konnte der junge Patient mit oraler Antibiose (875 mg Amoxicillin/125 mg Clavulansäure [Amoclav®]) bei reizloser Wunde sowie normwertigen Infektparametern aus der stationären Behandlung entlassen werden.

**Figure 5:**
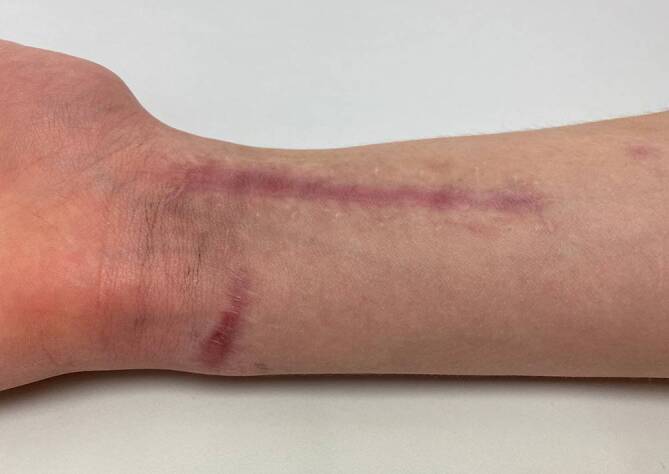

Die orale Antibiose wurde für insgesamt 6 Wochen durchgeführt. In den ambulant durchgeführten klinischen Kontrollen war die Beweglichkeit des Handgelenks zunächst noch eingeschränkt. Sechs Monate postoperativ zeigten sich hypertrophe, aber trockene Narben (Abb. 5). Weder klinisch noch laborchemisch bestand ein Anhalt auf einen Infekt (CRP 0,06 mg/dl [<0,05]; Leukozyten 3,66/nl [4,5–13]). Die frühere Beweglichkeit war fast vollkommen wieder hergestellt, lediglich bezüglich der Dorsalextension bestand noch ein Bewegungsdefizit von 5–10°.

## Diskussion

Anhand dieser Kasuistik soll die Schwierigkeit der Differenzialdiagnose zwischen einem intraossären Fremdkörper und einem Osteom an einem konkreten Fall dargestellt werden. Überdies soll der Leser ermutigt werden, externen Interpretationen von Röntgenbefunden stets mit einer gesunden Skepsis zu begegnen.

Die radiologische Differenzierung zwischen einem medullären Osteom und einem röntgendichten Fremdkörper ist nicht trivial. Das klassische Osteom ist ein Hamartom und kommt v. a. in den paranasalen Gesichtsschädelknochen vor. Daneben gibt es das medulläre Osteom, auch Enosteom oder Kompaktainsel genannt, welches im spongiösen Knochen meist als kleiner, bis zu 2 cm großer intramedullärer Zufallsbefund („giant osteoma“) auftritt.

**Figure 6:**
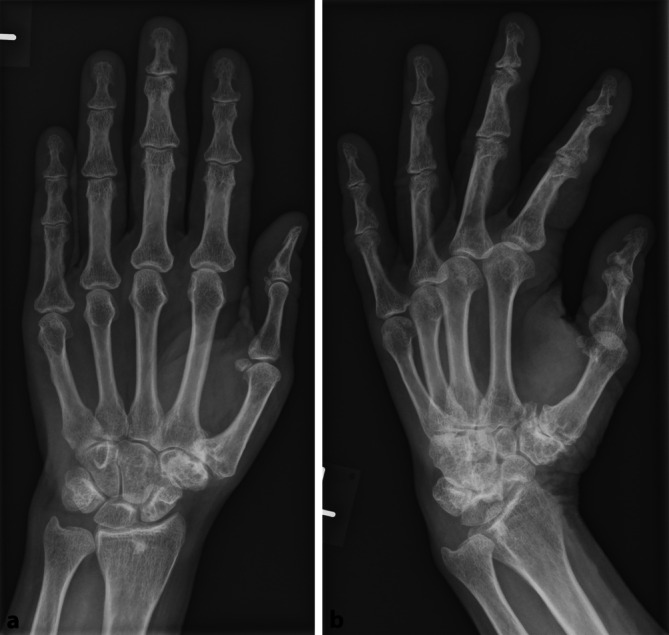

Ein Beispiel für ein an fast derselben Stelle liegendes medulläres Osteom bei einer 62-jährigen Patientin ohne Beschwerden zeigt Abb. 6a, b.

Das medulläre Osteom ist, wie auch ein Stein, charakteristischerweise scharf begrenzt. Anders als ein Stein besitzt es allerdings füßchenartige Ausläufer (Pseudopodien, [1]), die allerdings so fein sein können, dass sie im konventionellen Röntgenbild nicht sichtbar sind. Eine sichere Unterscheidung ist in diesem Fall aufgrund der auch zum intramedullären Osteom passenden Lage und Konfiguration des Steins nur durch die Dichtemessung einer CT-Untersuchung möglich. Als weitere Differenzialdiagnose für eine scharf begrenzte, röntgendichte Struktur gilt noch die osteoblastische Metastase, welche allerdings in diesem Fall aufgrund des Patientenalters nicht infrage kommt.

Mutmaßlich waren die intraossären Fremdkörper bereits durch den Erstbehandler als intramedulläres Osteom fehlinterpretiert worden. Der relevante Traumamechanismus sowie die stark verunreinigten Wunde direkt oberhalb der Fraktur hätten Anlass für eine gründliche Wundexploration sein müssen. Mit ihrem Vorgehen wichen die Kollegen erheblich von den üblichen Standards in der Frakturversorgung ab.

Vom Weiterbehandler wurde diese Fehlinterpretation leider unkritisch übernommen. Zweifelsohne ist die Weiterversorgung eines primär extern anatomisch reponierten und wundversorgten Patienten, ohne die Kenntnis des Primärbefundes, erheblich schwieriger. Bei kritischer Betrachtung hätten jedoch eine Weichteilverletzung direkt über der Fraktur sowie multiple Fremdkörper in den palmaren Weichteilen Anlass zur Skepsis und zur erweiterten Diagnostik sein müssen.

## Fazit für die Praxis

- Eine operative Wundexploration ist bei offenen Frakturen obligat. Bei primär nichterfolgter Exploration sollte diese zeitnah nachgeholt werden.
- Eine radiologische Differenzierung zwischen intraossären röntgendichten Fremdkörpern und medullären Osteomen ist mitunter schwierig.
- Ausschlaggebend für die korrekte Diagnose ist stets die Zusammenschau von Anamnese, klinischer Untersuchung und CT-Diagnostik.
- Externen Interpretationen von Röntgenbefunden sollte stets mit einer gesunden Skepsis begegnet werden.
